# A Distinct Type of Heterochromatin at the Telomeric Region of the *Drosophila melanogaster* Y Chromosome

**DOI:** 10.1371/journal.pone.0086451

**Published:** 2014-01-24

**Authors:** Sidney H. Wang, Ruth Nan, Maria C. Accardo, Monica Sentmanat, Patrizio Dimitri, Sarah C. R. Elgin

**Affiliations:** 1 Department of Biology, Washington University, St. Louis, Missouri, United States of America; 2 Dipartimento di Biologia e Biotecnologie “Charles Darwin” and Istituto Pasteur Fondazione Cenci-Bolognetti, Sapienza Università di Roma, Roma, Italy; Ludwig-Maximilians-Universität München, Germany

## Abstract

Heterochromatin assembly and its associated phenotype, position effect variegation (PEV), provide an informative system to study chromatin structure and genome packaging. In the fruit fly *Drosophila melanogaster*, the Y chromosome is entirely heterochromatic in all cell types except the male germline; as such, Y chromosome dosage is a potent modifier of PEV. However, neither Y heterochromatin composition, nor its assembly, has been carefully studied. Here, we report the mapping and characterization of eight reporter lines that show male-specific PEV. In all eight cases, the reporter insertion sites lie in the telomeric transposon array (*HeT-A* and *TART-B2* homologous repeats) of the Y chromosome short arm (Ys). Investigations of the impact on the PEV phenotype of mutations in known heterochromatin proteins (i.e., modifiers of PEV) show that this Ys telomeric region is a unique heterochromatin domain: it displays sensitivity to mutations in HP1a, EGG and SU(VAR)3-9, but no sensitivity to *Su(z)2* mutations. It appears that the endo-siRNA pathway plays a major targeting role for this domain. Interestingly, an ectopic copy of *1360* is sufficient to induce a piRNA targeting mechanism to further enhance silencing of a reporter cytologically localized to the Ys telomere. These results demonstrate the diversity of heterochromatin domains, and the corresponding variation in potential targeting mechanisms.

## Introduction

Heterochromatin represents a unique type of chromatin structure that confers transcriptional silencing by regular packaging of distinct domains enriched for repetitious DNA [1]. Abnormalities in the formation and/or maintenance of heterochromatin therefore are commonly associated with transposon activation and genome instability [2]. Heterochromatin also plays an important role in cell division; as part of the centromeric structure, heterochromatin is required for proper segregation of chromosomes during mitosis [3]. Other regulatory roles of heterochromatin, such as telomere length homeostasis [4], and proper expression of heterochromatic genes [5], have also been documented.

The Position Effect Variegation (PEV) phenotype, commonly monitored in the adult fly eye, has been used in many previous studies as an indicator of the degree of heterochromatin formation at the underlying locus [6]. PEV results from positioning a euchromatic reporter gene in or close to a heterochromatic environment by transposition or rearrangement. Several lines of evidence indicate that the spreading of heterochromatin packaging into the promoter region of the euchromatic gene is the cause of transcriptional silencing [7]. Silencing of the underlying euchromatic gene occurs in some but not all cells in a population, giving rise to a variegating phenotype; this differential spreading of heterochromatin is suggested to be a stochastic process [8]. The extent of silencing (variegation) can serve as a proxy for the extent of heterochromatin formation at the particular locus. A mutation that impacts the level of PEV is therefore indicative of a gene that functions in the formation and/or maintenance of heterochromatin [9]. Identification of mutations resulting in strong suppression of PEV (loss of silencing) and molecular characterization of these *Su(var)* loci has laid the groundwork for understanding heterochromatin formation in flies [10], [11]. Genes such as *Su(var)3-9* (a histone H3K9 methyltransferase) and *Su(var)3-3* (an H3K4 demethylase), identified and characterized under this paradigm, have revealed much of what we know about this alternative chromatin state [6].

PEV has been used as an assay to probe the heterochromatic landscape of the genome [12]. A P-element harboring an *hsp70-white* reporter gene was mobilized in the fly genome to identify heterochromatic regions, those that induce a variegating eye phenotype [12]. This screen recovered lines with insertions into pericentric domains, telomere associated satellite-like (TAS) regions, the fourth chromosome and the Y chromosome, as anticipated from prior cytogenetic analysis. Thus it produced PEV reporter lines that can be used to monitor the structure of heterochromatin across the genome. Use of these lines quickly established that not all heterochromatin has the same composition; different domains show distinct responses to different *Su(var)* mutations [13], [14]. Based on the differential response profile to well-known suppressors of variegation (as well as other evidence), a major distinction has been made between pericentric and telomeric heterochromatin, suggesting that different assemblies and silencing mechanisms are involved [15]. In particular, telomere position effect (TPE; studied using reporters in the subtelomeric TAS elements) is inert to mutations in *Su(var)205* (which codes for HP1a) but is suppressed by mutations in *Su(z)2* (a component of the Pc system) [13], [15], while the opposite is true for pericentric PEV. Further analyses have revealed additional unique domains of heterochromatin in the genome. For example, fourth chromosome PEV is not generally suppressed by mutations in *Su(var)3-9* [14], but is sensitive to mutations in *egg*, indicating that a different histone methyltransferase (HMT) is required for silencing [16]–[18]. Additional analyses of this type are likely to reveal more distinct types of heterochromatin, presumably reflecting differences in the underlying DNA repeat sequences and their organization.

The question of how different types of heterochromatin are established in the genome remains an active area of research. Different targeting mechanisms could be utilized for different types of heterochromatic domains. In flies, both the piRNA pathway and the endo-siRNA pathway have been implicated in targeting heterochromatin formation [19]–[21]. While these studies provide evidence supporting a small RNA targeting model for heterochromatin formation at some repetitious elements, given the diversity of heterochromatin domains, one can also anticipate a diversity of targeting mechanisms.

In flies, the Y chromosome has long been known to be a largely heterochromatic domain. In fact, Y chromosome dosage was one of the first modifiers of PEV to be identified [22]. The Y has been described as a ‘sink’ for components essential for heterochromatin formation and/or maintenance. It appears that additional copies of the Y chromosome impact PEV at other loci in the genome by competing for a limited amount of shared key factors required for heterochromatin integrity [23], [24]. Polymorphisms in the Y-linked rDNA loci have also been shown to modulate the heterochromatic landscape of the genome [25]. Recently, reports from Hartl and colleagues have further elaborated on how polymorphisms in Y chromosome heterochromatin can impact chromatin-based regulatory processes in other regions of the genome [26]–[28]. However, relatively little is known about the formation/maintenance of heterochromatin on the Y chromosome itself. A paucity of PEV reporter lines for the Y chromosome is one of the major obstacles in studying the mechanisms involved in heterochromatin formation and maintenance within this domain.

Over the past decade, we have collected eight variegating lines exhibiting a male-specific PEV phenotype. Here we map the insertion sites of these eight lines to the telomeric transposon array (*HeT-A* and *TART-B2* repeats) of Ys (short arm of the Y chromosome). We further characterize the heterochromatic properties of this region by examining the impact of mutations in PEV modifiers on these reporters. This telomeric Ys heterochromatin shows a unique response profile compared to other parts of the genome; nonetheless, our studies suggest that some of the mechanisms for heterochromatin formation and maintenance are shared among the Y chromosome, pericentric, and 4^th^ chromosome heterochromatins. While it appears that the endo-siRNA pathway is likely the major mechanism used to target heterochromatin formation at this domain, an ectopic copy of the *1360* transposon remnant is sufficient to drive a piRNA-dependent heterochromatin targeting mechanism to further enhance silencing of a reporter cytologically localized to the Ys telomere. We conclude that the Ys telomeric region is a unique domain of heterochromatin. Further investigation of this region will be informative in understanding chromatin packaging in general.

## Results

**Figure 1:**
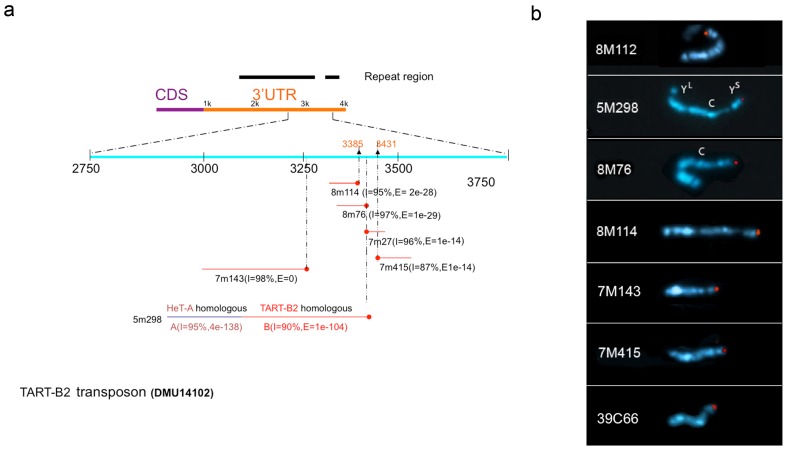
Sequencing and *in situ* hybridization mapping locate the Y-linked PEV reporters in the telomeric transposon arrays of Ys. (a) Alignment of reporter insertion flanking sequences to a consensus sequence of telomeric retro-transposon *TART-B2*. The 1 Kb region in the 3′-UTR harboring the insertion sites is magnified. The effective sequence read length for each reporter line is represented by the red line aligned to the region. The red dot at the end of each red line indicates the 5′ end of the reporter. The reporter flanking sequence of line 5m298 starts in the 3′ end of a *TART-B2* element and extends upstream to a neighboring *HeT-A* element, suggesting insertion into a partial fragment of *TART*. (b) *In situ* hybridization images of the metaphase chromosomes from third instar larval brain squashes. Only the Y chromosome of the representative metaphase spread is shown. DAPI staining is pseudo-colored in blue and the hybridization signal is in red. (C: centromere, Y^L^: long arm, Y^S^: short arm).

To precisely locate the insertion sites of the Y-linked PEV reporters, we performed inverse PCR followed by sequencing. While we cannot precisely map the location of each insert using BLASTN against the entire genome assembly, we can nonetheless map the insertion sites for all eight Y-linked reporter lines to internal regions of the telomeric retrotransposons. In six of these lines the reporter element is inserted into a *TART-B2* element, while in the other two lines (39C66 and 8M112) the reporter is inserted into a *HeT-A* element (*HeT-A* subfamily D and *HeT-A* t*o HeT-A Junction* respectively). Surprisingly, all six reporters inserted into the *TART-B2* element are located in the 3′-UTR of the element, with five of them clustered within a 50 nts range when mapped back to a *TART-B2* consensus sequence (Fig. 1a). Because there are multiple copies of *TART-B2* elements on the Y chromosome, and the quality of this region of the published assembly is relatively poor, we cannot resolve in which *TART-B2* elements these insertions reside. However, a comparison of the flanking sequences among the six insertion lines identifies sequence polymorphisms, which suggests that the inserts are in different copies of *TART-B2*. This result suggests that there is a common region in the *TART-B2* 3′UTR that is a particular hotspot for P-element insertions, consistent with results previously described by Mason and colleagues [29].

*HeT-A* and *TART* elements are distributed throughout telomeric and pericentric regions of the Y chromosome [30]. To further distinguish between these potential insertion sites, we performed *in situ* hybridization on metaphase chromosomes using the reporter sequence as a probe. Interestingly, we found that in all eight lines the reporter is inserted at the tip of Ys (Fig. 1b). These observations allow us to conclude that all eight reporter lines characterized here have an insert in the telomeric terminal retrotransposon array of Ys. The cytological results are consistent with the molecular mapping results presented above – both indicate that this region of the Y chromosome is relatively accessible to P element insertions (Fig. 1). It should be noted that reporters inserted into the terminal retrotransposon array of *HeT-A, TAHRE*, and *TART* have been previously described for the major autosomes [29]. Most of these insertion lines do not show a variegating phenotype unless the reporter is located close to the TAS region [29]. We therefore infer that our variegating reporters likely reflect the results of a competition between the spreading of adjacent heterochromatin and the expression of these retrotransposons, which has created a unique heterochromatic domain.

**Figure 2:**
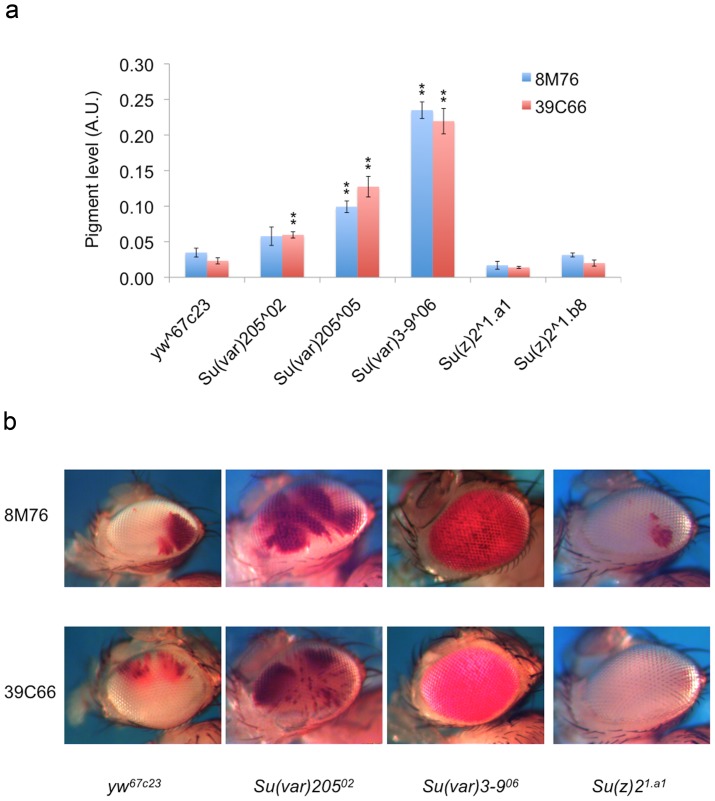
Response of Y-linked PEV reporter lines to mutations in well-known modifiers of PEV and TPE (dominant effects). (a) Pigment level quantification representing the level of PEV. Progeny from a cross with *yw^67c23^* is used as the wild type control. The allele used in each cross is shown on the X-axis. (Bars represent the average pigment level ± standard error. Asterisks are used to indicate mutant alleles that show statistically significant modifier activities, single, p<0.05; double, p<0.005.) (b) Representative pictures showing the dominant impact of the mutations on the fly eyes. The allele used for each modifier is listed below each column. The reporter line used is shown to the left of each row.

These variegating reporter lines present a new opportunity to elucidate the chromatin structure at a telomere of the Y chromosome. We first looked at the response of these variegating reporters to well-characterized modifiers of PEV and TPE (Fig. 2). Given that all of these reporter lines carry inserts in the *HeT-A* and *TART* elements of Ys, we chose two lines, 39C66 (a *HeT-A* insert) and 8M76 (a *TART* insert), as representatives of this set for further analysis. We first tested dominant effects of mutations in modifiers of TPE. Multiple alleles of *Su(z)2*, a transcription factor, have previously been shown to significantly suppress TPE of TAS inserts [13], [15]. However, no obvious suppression effects were observed for both of the alleles tested here (Fig. 2a, b), suggesting that the telomeric retrotransposon region of Ys does not have the chromatin structure typical for the TAS telomere-associated arrays, which are immediately upstream of *HeT-A* and *TART* arrays in the autosomes. We next examined the impact of a classic PEV suppressor, *Su(var)205*, on these reporters. *Su(var)205* encodes a chromo-domain containing protein, HP1a, which is implicated in the formation and spreading of heterochromatin [31]–[33]. Despite the known role of HP1a in telomere capping [34], [35], mutations in *Su(var)205* do not appear to modify TPE as seen using lines with the reporter inserted in TAS [13]. Interestingly, despite the fact that these inserts are found in the telomeric region, both *Su(var)205* alleles tested here show significant dominant suppression of variegation for both reporters (Fig. 2a, b). These observations suggest a response profile for the Ys *HeT-A/TART* telomeric heterochromatin that is more similar to PEV than TPE. We next tested the impact of an insertion mutation of *Su(var)3-9*. The *Su(var)3-9^06^* allele disrupts the production of the SU(VAR)3-9 protein [36] and has been shown to impact both TPE and PEV [15]. Strong dominant suppression of variegation is observed with this allele for these Y-linked reporters (Fig. 2a, b), indicating an important role for this gene product in the chromatin structure at this region.

**Figure 3:**
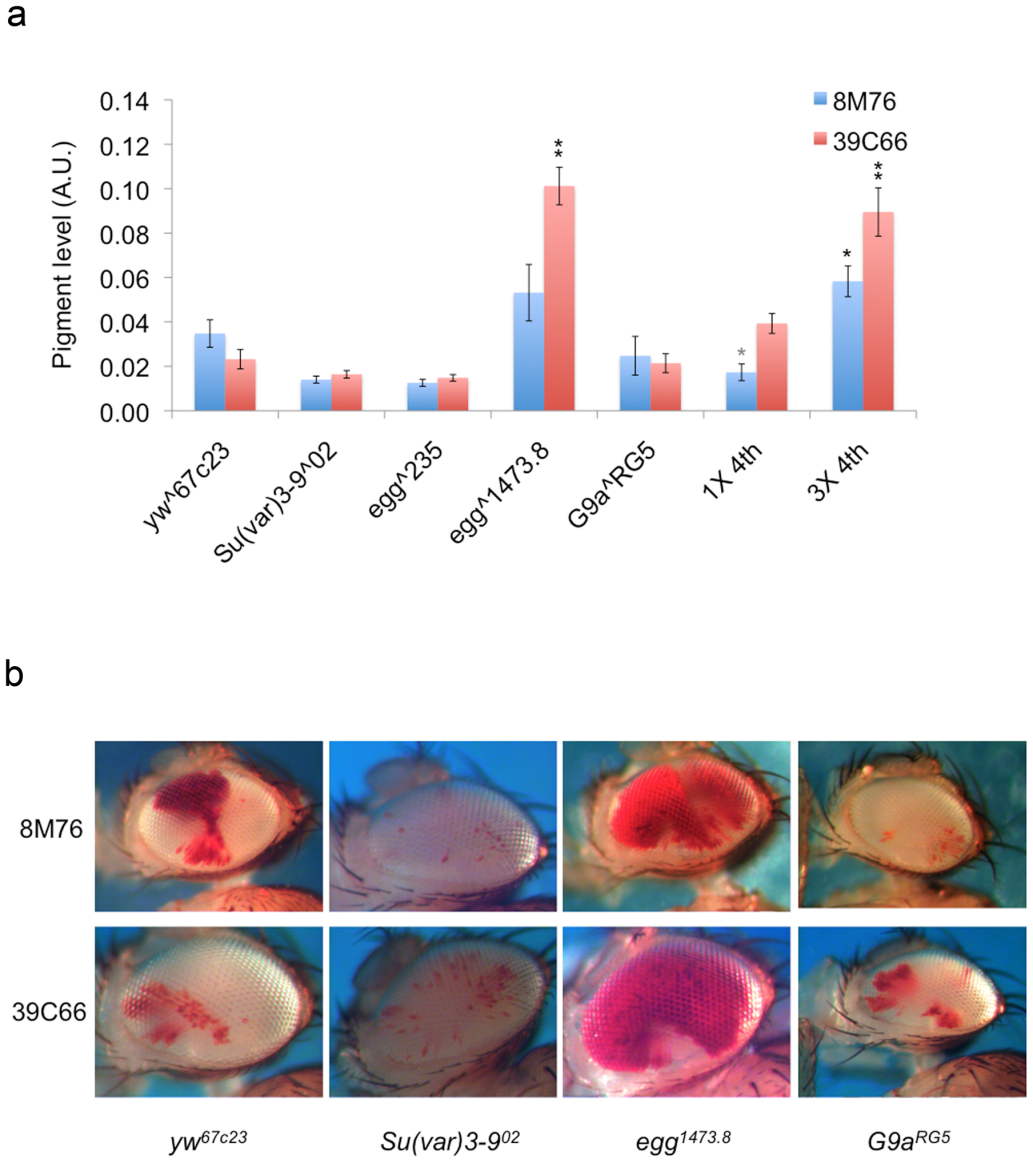
Impact of mutations in HMTs and of dosage of the 4^th^ chromosome on the level of variegation of the Y-linked reporters. (a) Pigment level quantification showing the level of expression. (Bars represent the average pigment level ± standard error. Asterisks are used to indicate mutant alleles that show statistical significant modifier activities, single, p<0.05; double, p<0.005.) 1X 4^th^ and 3X 4^th^ represent the copy number of the 4^th^ chromosome in the assayed flies. Note that the control line *yw^67C23^* has 2X 4^th^ (See Table S1 for information on the fly lines used.), the t test on 1X 4^th^ examines E(var) activities of the allele (see materials and methods for details). (b) Representative pictures showing the dominant impacts on PEV in the fly eye from mutations disrupting HMT activities.

SU(VAR)3-9 is a histone 3 lysine 9 methyl-transferase. Given the strong impact of the *Su(var)3-9^06^* allele on variegation, we reasoned that SU(VAR)3-9 might function through its enzymatic activity to modify the chromatin structure at this region. However, an allele disrupting the enzymatic activity of SU(VAR)3-9 [36], *Su(var)3-9^02^*, did not show a suppression of variegation (Fig. 3a, b). This suggests that the critical function of SU(VAR)3-9 in this region is structural rather than enzymatic. This interpretation is supported by previous results documenting the antipodal effect of SU(VAR)3-9 on PEV [23]. Our observations lead us to infer that SU(VAR)3-9 is likely not the HMT functioning in the Ys telomeric heterochromatin.

To identify the potential HMTs functioning at this region, we looked for effects from mutations in the genes for other known H3K9 HMTs. In addition to *Su(var)3-9*, two more genes, *egg* and *G9a*, have been identified in the fly genome as potential H3K9 HMTs [18], [37]–[39]. Dominant effects of mutations in *egg* and a recessive effect of *G9a* were tested for their impact on the variegation phenotype of these reporters. Only the *egg^1473.8^* allele shows a strong suppression of variegation at these sites, consistent with the interpretation that EGG is the major HMT functioning in the formation/maintenance of heterochromatin at the Ys telomeric region. The two *egg* alleles tested show different effects on the suppression of variegation. The *egg^1473.8^* allele is a deletion of the entire SET domain, a domain which is required for the HMT activity of EGG [40]. In contrast, the *egg^235^* allele has a di-nucleotide substitution that creates a cryptic splice site for the 4th intron [40]. Retention of this intron will introduce a premature stop codon that results in a protein product with no identifiable functional domains. However, a cryptic splice site actually allows normal splicing to occur at a low frequency, which results in the production of some wild type protein [40]. The comparison on the impact from the two *egg* alleles therefore represents a comparison between a dominant effect of the SET domain deletion and an incomplete null mutation. We interpret the discrepancy between the two alleles in their impact on variegation as an additional piece of evidence demonstrating the importance of the HMT activity of EGG in this region.

EGG has previously been characterized as a 4^th^ chromosome-specific HMT [16]–[18]. It is also known to impact expression from some reporters in the pericentric heterochromatin [17]. Nonetheless, the observations above suggest that the 4^th^ chromosome and Ys telomeric heterochromatin share common components for heterochromatin formation or maintenance. To test this hypothesis, we took advantage of the attached 4^th^ chromosome line [14] to generate flies with only one copy or with three copies of the 4^th^ chromosome to examine the impact of dosage on Ys telomeric heterochromatin. Increasing heterochromatic mass of a particular type in the genome could lead to increased competition for the available components for heterochromatin formation/maintenance [23]. On increasing dosage of the 4^th^ chromosome, we do observe a suppression of variegation for the Y-linked reporters (Fig. 3a), whereas previous studies have found no effect of 4^th^ chromosome dosage on reporters in either the pericentric or telomeric (TAS) regions of the 2^nd^ chromosome [14]. Earlier studies have shown that Y chromosome dosage does have a similar impact on fourth chromosome reporters [12]. These observations reinforce the conclusion that the 4^th^ chromosome and the Ys telomeric heterochromatin share some unique components for heterochromatin formation and/or maintenance.

**Figure 4:**
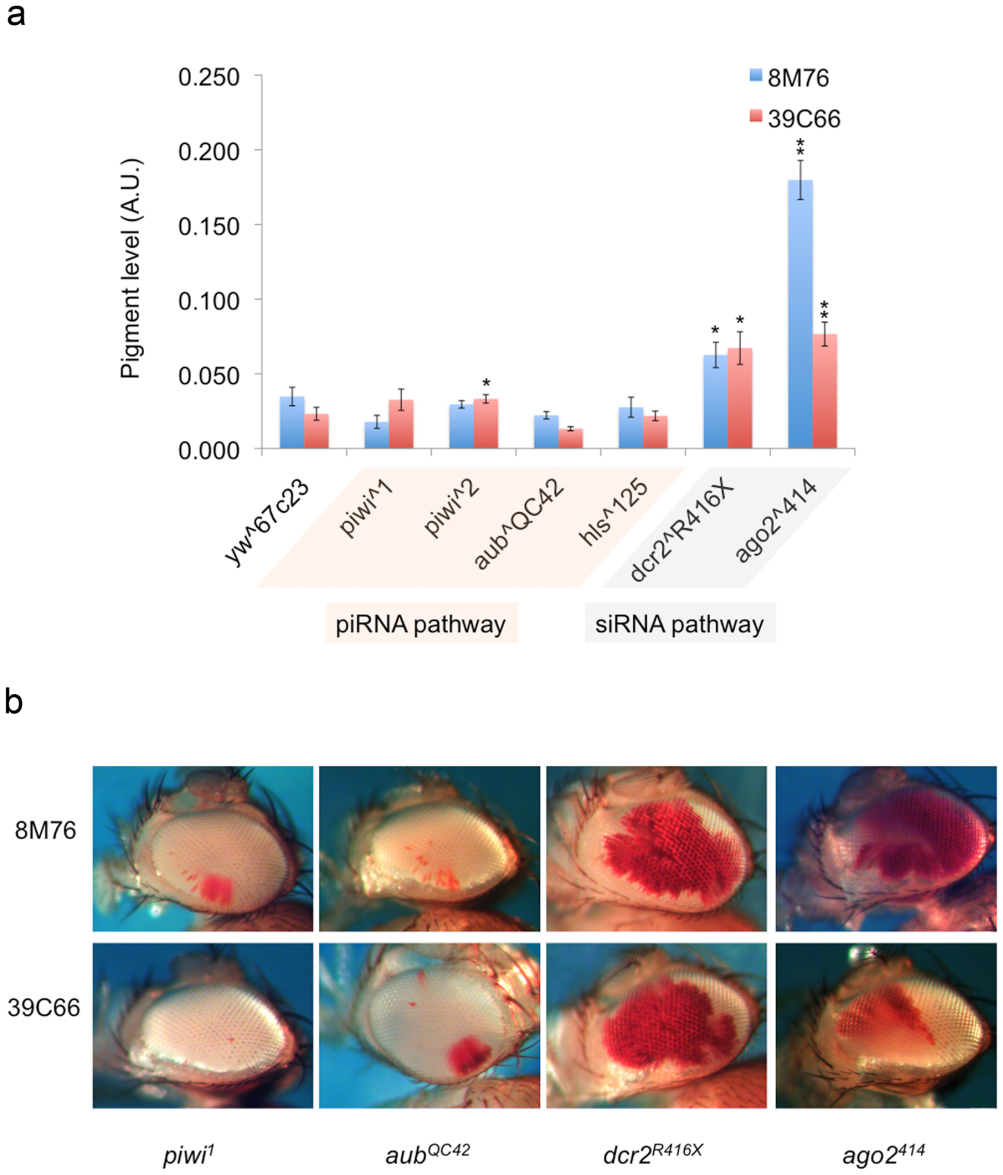
Impacts of mutations in components of the small RNA pathways on the level of variegation of the Y-linked reporters. (a) Pigment level quantification indicating the extent of the suppression of PEV. (Bars represent the average pigment level ± standard error. Asterisks are used to indicate mutant alleles that show statistical significant modifier activities, single, p<0.05; double, p<0.005.) Pathways requiring the genes tested are indicated below the allele names. (b) Representative pictures showing the dominant impacts on PEV in the fly eye from mutations disrupting small RNA pathways.

Small RNA targeting mechanisms have been demonstrated to be one of the major mechanisms for initiating the formation of heterochromatin in the fission yeast *S. pombe* [41]–[43]. In the fruit fly, both siRNA and piRNA systems have been implicated in this process [19]–[21]. To ask whether small RNA targeting of heterochromatin formation could participate in the formation of Ys telomeric heterochromatin, we examined the impacts of dominant mutations in both siRNA and piRNA pathways. No obvious impact is observed when mutations in components of the piRNA pathway are introduced (Fig. 4a, b). We examined the impacts of *piwi^1^*, *piwi^2^*, *aub^QC42^* and *hls^125^* mutations on the variegation of the Ys telomeric reporters, and no obvious suppression effects were observed, although there may be a weak effect of the *piwi^2^* allele on 39C66 (Fig. 4a). In contrast, both mutations in the siRNA pathway that were tested strongly suppress variegation of these reporters (Fig. 4a,b), indicating an involvement of this pathway in the heterochromatin silencing of the Ys chromosome. *Dcr-2^R416X^* has a point mutation that truncates the protein produced and disrupts its function in producing siRNA [44]. *ago2^414^* is a loss of function allele with its second exon deleted by imprecise excision [45]. That mutations in the siRNA pathway dominantly suppress variegation indicates a role for siRNA in targeting heterochromatin formation in this region.

**Figure 5:**
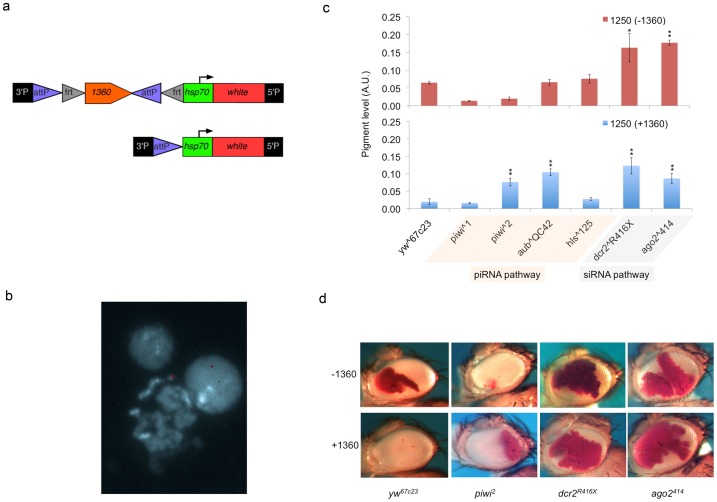
An ectopic *1360* element enhances Ys telomeric PEV via a piRNA-dependent mechanism. (a) Diagram showing the construct used in this line. FRT sites (gray triangles) flanking the ectopic copy of the *1360* element allow a FLPase mediated excision of the element. (b) *In situ* hybridization image of metaphase chromosomes of line 1250. The DAPI staining is pseudo-colored in blue and the hybridization signal in red. (c) Pigment level quantification comparing the impact on reporter expression of mutations in different small RNA pathway components with and without the ectopic copy of the *1360* element in the reporter. (Bars represent the average pigment level ± standard error. Asterisks are used to indicate mutant alleles that show statistical significant modifier activities, single, p<0.05; double, p<0.005.) (d) Representative pictures comparing dominant impacts on PEV in the fly eye from mutations disrupting small RNA pathways, with (+) and without (−) the ectopic copy of the*1360* element in the reporter.

Transposon density, particularly that of the *1360* DNA transposon, has been shown to be correlated with heterochromatin silencing on the 4^th^ chromosome [46]. Previously we have shown that introducing an extra copy of *1360* can enhance the variegation phenotype of a reporter in regions sensitive to mutations in small RNA pathways [47]. In an independent screen using a P element containing a copy of *1360* upstream of the *hsp70-white* reporter (Fig. 5a), we recovered an additional Y-linked PEV line, line 1250 [48]. The *1360* element in this construct is flanked by FRT sites, which allows FLP-mediated excision to test its impact on PEV (Fig. 5a). We were unable to precisely map the insertion site of line 1250 using inverse PCR sequencing. However, *in situ* hybridization experiments map the insertion site again in the telomeric region of Ys (Fig. 5b). Reporter line 1250 therefore provides an opportunity to examine the sensitivity of variegation in the Ys telomeric region to an ectopic *1360* element. FLP-mediated excision of the *1360* element resulted in strong suppression of variegation at this locus (Fig. 5c, d; *yw^67c23^*, compare +*1360* with −*1360*), consistent with the interpretation that the exogenous *1360* element plays an important role in promoting local heterochromatin structure. We next investigated the potential mechanism of this *1360*-dependent enhancement of heterochromatin silencing. We examined the impact of mutations in the siRNA and piRNA pathways on the variegation phenotype in reporter lines 1250 with and without the extra copy of the *1360* element. In the absence of the extra *1360* element, the reporter line 1250 shows similar responses to mutations in components of the small RNA pathways as seen for the other lines tested in this study (compare Fig. 4a, 5c). Interestingly, with the ectopic copy of *1360* element present, the same reporter appears to show dominant suppression of variegation in response to mutations of *piwi* and *aub* (Fig. 5c). This suggests that the enhancement of variegation resulting from the extra copy of the *1360* element is operating via a piRNA dependent targeting mechanism. We therefore conclude that the ectopic copy of the *1360* element at the Ys telomeric region is sufficient to recruit the piRNA-dependent targeting machinery to enhance heterochromatin silencing in a region that is normally dependent on the siRNA pathway for heterochromatin targeting.

## Discussion

Despite being one of the first heterochromatic regions in the fly genome to be identified, the packaging of Y chromosome is not well understood. The poor quality of the sequence assembly in this region of the fly genome severely restricts our ability to perform a comprehensive survey of its chromatin landscape. Reporter insertion lines that can be uniquely mapped by *in situ* hybridization therefore present unique opportunities to explore the chromatin packaging of this region.

Screens using a P element carrying an *hsp70-white* reporter have led to the recovery of variegating lines with an insertion into the *HeT-A/TART* arrays at the Ys telomere. This is surprising, in that the *HeT-A/TART* retrotransposons are know to be expressed, and prior studies [29] have reported that insertions into similar telomeric sequences in the autosomes results in full expression of the reporter, unless the reporter is positioned close to the proximal TAS arrays, which are silenced. We have analyzed the impacts on the observed PEV phenotype of our Ys telomere reporters resulting from mutations in PEV modifiers that are well characterized. We have found that these reporters do not mimic the TAS-associated TPE reporters seen on the autosomes; instead, the Ys reporters show strong suppression in response to mutations in the gene for HP1a, and no suppression in response to mutations in *Su(z)2*. In addition, we found that while the chromatin structure at this region is sensitive to the dosage of Su(var)3-9 (in contrast to the 4^th^ chromosome heterochromatin), it actually requires the SET domain of EGG for proper silencing (similar to the 4^th^ chromosome). These results enable us to conclude that the telomeric *HeT-A/TART* region of Ys is a unique domain of heterochromatin. Whether this reflects a structure uniquely targeted to these Y chromosome *HeT-A* and *TART* elements, or the spreading of a heterochromatin structure targeted to other adjacent repetitious elements, cannot be determined given the current information. Regardless, we propose that the Ys telomeric region should be added to the list of distinct subcategories of heterochromatin. While each of these domains has unique characteristics, they nonetheless appear to share certain modifiers and utilize some of the same proteins for heterochromatin formation.

Given the repetitive nature of Y chromosome, we propose a targeting mechanism for its heterochromatin formation that utilizes small RNAs derived from transposable elements. We have found that while reporters in this region normally respond to mutations in the endo-siRNA pathway, an ectopic copy of the*1360* element is sufficient to enhance heterochromatic silencing via a piRNA-dependent silencing pathway. This observation again suggests complex cross talk between different mechanisms of heterochromatin targeting/formation. It should be noted, however, that while we were able to map the insertion site for line 1250 to the Ys telomeric region using *in situ* hybridization, we were unable to align the flanking sequence to any consensus sequence. This observation indicates that the reporter construct for line 1250 is not located within a known transposon, which is in contrast to the rest of the reporter lines tested in this study. The significance of this difference remains to be investigated.

Our identification of an additional type of heterochromatin corroborates the multiple chromatin states models resulting from large-scale genome-wide studies of the distribution of histone modifications and chromosomal proteins, such as modENCODE [49]. As our study demonstrates, while the heterochromatin/euchromatin dichotomy is useful and convenient in describing much of what we know about chromatin structure, it is inadequate in capturing the diversity of chromatin structures within a genome. Future studies on the Y chromosome heterochromatin will likely yield new insights on the process of chromatin packaging and gene regulation.

## Materials and Methods

### Fly Stocks, Genetics and Husbandry

Fly lines 39C66, 5M298, 7M27, 7M143, 7M415, 8M76, 8M112, 8M114 and 1250, were recovered from transposition-based screens that have been previously reported [12], [48], [50]. Crosses testing for a dominant effect of known *Su(var)s* were carried out at 25°C, 70% humidity on regular cornmeal sucrose-based medium [51]. In each cross, male flies exhibiting a representative eye phenotype for a given reporter line were crossed to female virgins carrying the specified modifier mutation. The 3X 4^th^ line has one copy of the normal 4^th^ chromosome and one copy of the attached 4^th^ chromosome. More detailed information on modifier lines used is listed in Table S1. Standard balancers are used to maintain the mutation in each stock.

### Inverse PCR and Sequencing

Inverse PCR to amplify the region flanking the insertion site was done as previously described [46]. The PRC product was than treated with ExoSAP (Affymetrix) and sequenced using BigDye Terminator v1.1 (Applied Biosystems) following vendor’s instructions. The sequence results were then analyzed using NCBI BLAST with the nr database.

### *In situ* Hybridization

*In situ* hybridization on metaphase chromosomes from third instar larval neuroblasts was done as previously described [52]. The probe used in this study was the P element reporter containing *hsp26-pt* and an *hsp70*-driven *white* gene [12].

### PEV Assay

Ethanol based pigment extraction and quantification was essentially done as previously described [46] with some minor adjustments. The overnight incubation step at 4°C was omitted. To increase the throughput and consistency, a Mixer Mill MM 300 was utilized to homogenize the sample and a plate reader was used for spectroscopy. For each genotype, three to five samples were measured for pigment level; each sample is composed of five male flies (3∼5 days old) randomly selected from the population.

### Statistical Analysis

The statistical significance of the impact of a given mutant allele on the PEV eye phenotype was assessed by performing one-sided Welch two-sample t tests for multiple independent samples. In each case [except for the haploid 4^th^ mutant line] the significance of the increase in red pigmentation (suppression of variegation) relative to the *yw* background was tested at levels of p<0.05 and p<0.005. The Enhancer of variegation [*E(var*)] activity of the haploid 4^th^ mutant was assessed in the same way, except that a decrease in pigmentation is expected and tested accordingly. All tests were performed with the R statistical programming language [53].

## Supporting Information

Table S1 The genotypes of all fly lines used in this study are shown (middle column), with the source (lab and pertinent reference) given (right-hand column). If the stock is available from the Bloomington Stock Center, the stock number is provided (left-hand column).
(DOCX)
