# Supplementary material for: A Distinct Type of Heterochromatin at the Telomeric Region of the Drosophila melanogaster Y Chromosome
Source: PLoS One. 2014 Jan 24;9(1):e86451. doi: 10.1371/journal.pone.0086451 (PMC3901700; doi:10.1371/journal.pone.0086451)
Supplement: Table S1 — The genotypes of all fly lines used in this study are shown (middle column), with the source (lab and pertinent reference) given (right-hand column). If the stock is available from the Bloomington Stock Center, the stock number is provided (left-hand column). (DOCX) [file pone.0086451.s001.docx]

Table S1 List of fly lines used in this study

| Stock NO. | Genotype | Source |
| --- | --- | --- |
| NA | ago2^414^ | Siomi lab [1] |
| BL4968 | [w^1118^](http://flybase.org/reports/FBal0018186.html); [aub^QC42^](http://flybase.org/reports/FBal0030533.html) [cn^1^](http://flybase.org/reports/FBal0001731.html) [bw^1^](http://flybase.org/reports/FBal0001342.html)/[CyO](http://flybase.org/reports/FBba0000025.html), [P{sevRas1.V12}FK1](http://flybase.org/reports/FBti0000937.html) | Bloomington Stock Center |
| BL1785 | C(4)RM, ci[1] ey[R]/0 | Bloomington Stock Center |
| NA | yw; dcr2^R416X^ / CyO | Carthew lab [2] |
| BL30569 | w^*^; [bw^1^](http://flybase.org/reports/FBal0001342.html) [egg^1473^](http://flybase.org/reports/FBal0190397.html)/[SM1](http://flybase.org/reports/FBba0000037.html) | Bloomington Stock Center |
| BL30566 | w^*^; [bw^1^](http://flybase.org/reports/FBal0001342.html) [egg^235^](http://flybase.org/reports/FBal0212302.html)/[SM1](http://flybase.org/reports/FBba0000037.html) | Bloomington Stock Center |
| NA | G9a^RG5^ | Spierer lab [3] |
| NA | hls^Δ125^ | Birchler lab [4] |
| NA | yw; Su(var)205^02^ / CyO | Lab stock [5] |
| NA | yw; Su(var)205^05^ / CyO | Lab stock [5] |
| NA | yw; Su(var)3-9^02^ / TM3-sb | Reuter lab [6] |
| NA | w; Su(var)3-9^06^ / TM3-sb | Reuter lab [6] |
| BL5549 | Su(z)2^1.a1^/CyO | Bloomington Stock Center |
| NA | Su(z)2^1.b8^/CyO | Pirotta lab [7] |
| NA | w; piwi^1^/ CyO | Lin lab [8] |
| NA | w; piwi^2^/ CyO | Lin lab [8] |
| BL6599 | y^1^w^67c23^ | Bloomington Stock Center |

1. Okamura K, Ishizuka A, Siomi H, Siomi MC (2004) Distinct roles for Argonaute proteins in small RNA-directed RNA cleavage pathways. Genes & Development 18: 1655–1666.

2. Lee YS, Nakahara K, Pham JW, Kim K, He Z, et al. (2004) Distinct roles for *Drosophila* Dicer-1 and Dicer-2 in the siRNA/miRNA silencing pathways. Cell 117: 69–81.

3. Seum C, Bontron S, Reo E, Delattre M, Spierer P (2007) *Drosophila* G9a is a nonessential gene. Genetics 177: 1955–1957. doi:10.1534/genetics.107.078220.

4. Pal-Bhadra M, Leibovitch BA, Gandhi SG, Rao M, Bhadra U, et al. (2004) Heterochromatic silencing and HP1 localization in *Drosophila* are dependent on the RNAi machinery. Science 303: 669–672. doi:10.1126/science.1092653.

5. Hayashi S, Ruddell A, Sinclair D, Grigliatti T (1990) Chromosomal structure is altered by mutations that suppress or enhance position effect variegation. Chromosoma 99: 391–400.

6. Schotta G, Ebert A, Dorn R, Reuter G (2003) Position-effect variegation and the genetic dissection of chromatin regulation in *Drosophila*. Semin Cell Dev Biol 14: 67–75.

7. Cryderman DE, Morris EJ, Biessmann H, Elgin SC, Wallrath LL (1999) Silencing at *Drosophila* telomeres: nuclear organization and chromatin structure play critical roles. EMBO J 18: 3724–3735. doi:10.1093/emboj/18.13.3724.

8. Cox DN, Chao A, Baker J, Chang L, Qiao D, et al. (1998) A novel class of evolutionarily conserved genes defined by piwi are essential for stem cell self-renewal. Genes Dev 12: 3715–3727.
